# Establishment of an MRI‐Based Diagnostic Approach to Identify Critical Pulpal Inflammation Zones in a Rat Caries‐Derived Pulpitis Model

**DOI:** 10.1111/iej.70151

**Published:** 2026-03-29

**Authors:** Kiichi Moriyama, Motoki Okamoto, Masakatsu Watanabe, Hailing Huang, Koki Nakatani, Shigeyoshi Saito, Yusuke Takahashi, Mikako Hayashi

**Affiliations:** ^1^ Department of Restorative Dentistry and Endodontology, Graduate School of Dentistry The University of Osaka Osaka Japan; ^2^ Department of Oral Science and Translational Research, College of Dental Medicine Nova Southeastern University Fort Lauderdale Florida USA; ^3^ Department of Stomatology Aviation General Hospital of Beijing Beijing China; ^4^ Department of Medical Physics and Engineering, Division of Health Sciences, Graduate School of Medicine The University of Osaka Osaka Japan

**Keywords:** irreversible pulpitis, MRI, pulp biology, pulp diagnosis, pulpotomy, reversible pulpitis

## Abstract

**Aim:**

To establish a magnetic resonance imaging (MRI)‐based diagnostic approach for identifying ‘critical pulpal inflammation’ zones in a rat model of caries‐derived pulpitis.

**Methodology:**

Caries‐derived pulpitis was induced in the molars of Sprague–Dawley rats using the caries‐derived pulpitis model. T2‐weighted images by 7 Tesla MRI and micro‐computed tomography (CT) images were taken weekly to sequentially monitor the caries progression and pulp conditions in the same individuals. Caries lesions were classified into moderate, severe and exposed groups with reference to the micro‐CT images. Sound teeth without caries were used as controls. Histopathological observations were performed to confirm the pulp inflammation. Pulpotomy was performed to remove the low magnetic resonance (MR) signal area suspected to be a ‘critical pulpal inflammation’ zone, which is considered unlikely to be preserved. Six weeks after pulpotomy, T2‐weighted and micro‐CT images of the molars were obtained, and histopathological evaluation was performed to observe the outcome of the pulpotomy.

**Results:**

High MR signal areas expanded with caries progression from the moderate to the severe group, indicating a spreading inflammatory response, which was confirmed histologically. The exposed group showed a significantly lower MR signal area compared with the others (one‐way analysis of variance, Tukey's post hoc test, *p* < 0.05). Matrix Metalloproteinase 9 (MMP9), a candidate molecular biomarker of serious pulpal inflammation, and inflammatory cell infiltration were observed in the same area. After pulpotomy, the remaining pulp tissue showed tertiary dentine formation without pulp necrosis and absence of inflammatory markers and MMP9, suggesting successful removal of critical pulpal inflammation.

**Conclusions:**

This study suggests the potential of MRI as a novel non‐invasive and quantitative diagnostic method for critical pulpal inflammation zones. Moreover, MMP9 was identified as a reliable biomarker of critical pulpal inflammation. Future studies will focus on validating the potential biomarker role of MMP9 and analysing low MR signal areas for other novel biomarkers of critical pulpal inflammation.

## 1 Introduction

Dental caries, pulpitis and apical periodontitis remain prevalent and unresolved diseases in clinical dentistry worldwide (Listl et al. 2015; World Health Organization 2022). When deep caries invading the pulp tissue is diagnosed as being associated with serious pulpitis after clinical examination, pulpectomy, that is, removal of the entire pulp tissue, is generally indicated as a standard treatment. However, removal of the entire pulp may not only result in the loss of pulpal function of the protective response to external stimuli but also increase the risk of apical periodontitis or root fracture, ultimately leading to tooth loss (Axelsson et al. 2004; Kawahara et al. 2020). The number of remaining teeth is closely related to a healthy life expectancy (Matsuyama et al. 2017), and tooth loss may be associated with cerebrovascular disorders (Cheng et al. 2018), cardiovascular disturbances (Cheng et al. 2018; Peng et al. 2019), bone fractures (Wakai et al. 2013; Yu et al. 2021; Taguchi et al. 2023) frailty and dementia (Fang et al. 2018; Oh et al. 2018). Therefore, tooth and pulp preservation is important to reduce the risk of various systemic diseases and extend healthy life expectancy.

Preserving pulp vitality has become increasingly important in recent years (Duncan et al. 2019; American Association of Endodontists 2021). Pulpotomy, or partial pulp tissue removal, has attracted more attention (Cushley et al. 2019; Donnelly et al. 2022; Duncan 2022; Philip and Suneja 2022) than pulpectomy because of its minimally invasive nature. Pulpotomy selectively removes only the pulp tissue with serious inflammation (Cvek 1978) and is performed mainly in deciduous teeth. However, recent evidence indicates that pulpotomy has been successfully performed in permanent teeth that were traditionally considered to require pulpectomy due to serious pulpal inflammation (Ng et al. 2007; Asgary et al. 2014, 2018; Linsuwanont et al. 2017; Taha and Khazali 2017; Cushley et al. 2019; Uesrichai et al. 2019; Ather et al. 2022). This is because of the widespread use of dental microscopes, which provide a magnified field of view for treatment and the development of contemporary dental materials (Parirokh and Torabinejad 2010; Torabinejad and Parirokh 2010; Hosoya et al. 2019). Compared with direct pulp capping, which preserves the entire pulp tissue, pulpotomy involves a more reliable removal of caries‐infected dentine and irreversibly inflamed pulp tissue (Fuks et al. 1982) and tight restoration using dental materials to prevent postoperative bacterial leakage (Hecova et al. 2010; Donnelly et al. 2022). While performing pulpotomy, the critical pulpal inflammation zones, exhibiting extremely serious inflammatory responses unlikely to permit pulpal healing, must be accurately identified and completely removed (Ricucci et al. 2019). However, it is difficult to accurately delineate the area and degree of inflammation of the pulp preoperatively using current pulp examination methods, such as the electric pulp test and thermal sensitivity test (American Association of Endodontists 2021). The results of these methods are based on the pain sensation reported by the patient and vary greatly among individuals. Furthermore, these methods have a low correlation with the actual histopathological condition of the pulp (Mejàre et al. 2012). In addition, recent evidence suggests that traditional binary classification into ‘reversible’ and ‘irreversible’ forms, based primarily on clinical symptoms and the results of current pulp examination methods, lacks biological precision and does not reliably predict pulpal healing potential (Mejàre et al. 2012; Donnermeyer et al. 2023). A recent systematic review has demonstrated that teeth clinically diagnosed with the so‐called irreversible pulpitis may still respond favourably to vital pulp therapy, thus questioning the validity of this terminology (Asgary and Eghbal 2023). Consequently, conventional diagnostic results can serve only as supportive information, and at present, the actual clinical condition of the pulp is largely determined during the treatment procedure by direct visualization of the pulp tissue. However, only specialized and experienced dentists can differentiate between the pulp tissue requiring removal and the remaining tissue with healing potential, making pulpotomy a highly specialized and challenging treatment option in endodontics (Edwards et al. 2021). This is especially evident because of a poor understanding of the pathogenesis of pulpitis (Duncan et al. 2019). If an objective pulpal diagnosis can be accurately obtained pre‐ or intraoperatively, the success rate of pulpotomy is likely to increase. Therefore, novel pulpal examination methods need to be developed for a more accurate diagnosis of pulpal conditions.

Magnetic resonance imaging (MRI), a diagnostic imaging modality mainly used for soft tissue, is widely utilized in the medical and dental fields (Bartusik‐Aebisher et al. 2022; Flügge et al. 2023). It could be an effective and novel pulpal diagnostic tool; in fact, it can be used to determine pulp viability (Gahleitner et al. 1999; Idiyatullin et al. 2011; Assaf et al. 2014, 2015; Flügge et al. 2016; Cankar et al. 2020; Juerchott et al. 2022). However, most of the reports on pulpal diagnosis were based on clinical images of patients, and no reports have systematically analysed pulpitis in experimental animal models with a high reproducibility. In recent years, the upper limit of the static magnetic field of MRI has been approved for use in many countries, and more detailed images, mainly used for cerebrovascular diseases, can be obtained using the newly developed 7 Tesla (7T) ultra‐high magnetic field MRI, contributing greatly to improved diagnostic accuracy (Bubrick et al. 2022; Cheng et al. 2023; Clarke et al. 2023). The 7T MRI has a static magnetic field strength that is several times stronger than that of conventional MRI; therefore, the signal‐to‐noise ratio does not decrease even when the slice thickness of the imaging section is reduced, and detailed images of small areas can be captured (Regatte and Schweitzer 2007). Accordingly, we hypothesized that ultra‐high magnetic field MRI would enable a detailed observation of pulpitis in small animals, which is difficult using conventional MRI.

We previously established a rat caries‐derived pulpitis animal model based on caries progression using micro‐computed tomography (CT) and histopathological evaluation (Huang et al. 2023). However, accurate preoperative determination of the presence and critical pulpal inflammation zones, considered unlikely to be preserved, remains difficult. Based on these facts, a more detailed pathogenesis of caries‐derived pulpitis could be elucidated using a rat caries‐derived pulpitis model (Huang et al. 2023), 7T MRI (Regatte and Schweitzer 2007) and histopathological observations. In this study, we aimed to establish a basis for a novel diagnostic method for caries‐derived pulpitis using 7T MRI and a rat caries‐derived pulpitis model. This diagnostic method is expected to potentially increase the success rate of vital pulp therapy, including pulpotomy.

## 2 Materials and Methods

### 2.1 Study Approval

All animal studies were approved by the Institutional Animal Care and Use Committee of the Osaka University Graduate School of Dentistry (No. R‐01‐017‐0). The manuscript of this animal study has been written according to the Preferred Reporting Items for Animal studies in Endodontology (PRIASE) 2021 guidelines (Nagendrababu et al. 2021). Daily care, monitoring and welfare assessments were performed by qualified animal facility staff trained according to institutional and national regulations.

### 2.2 Sex as a Biological Variable

We only included male rats because male animals usually exhibit less variability in phenotype.

### 2.3 Rat Caries‐Derived Pulpitis Model

**FIGURE 1:**
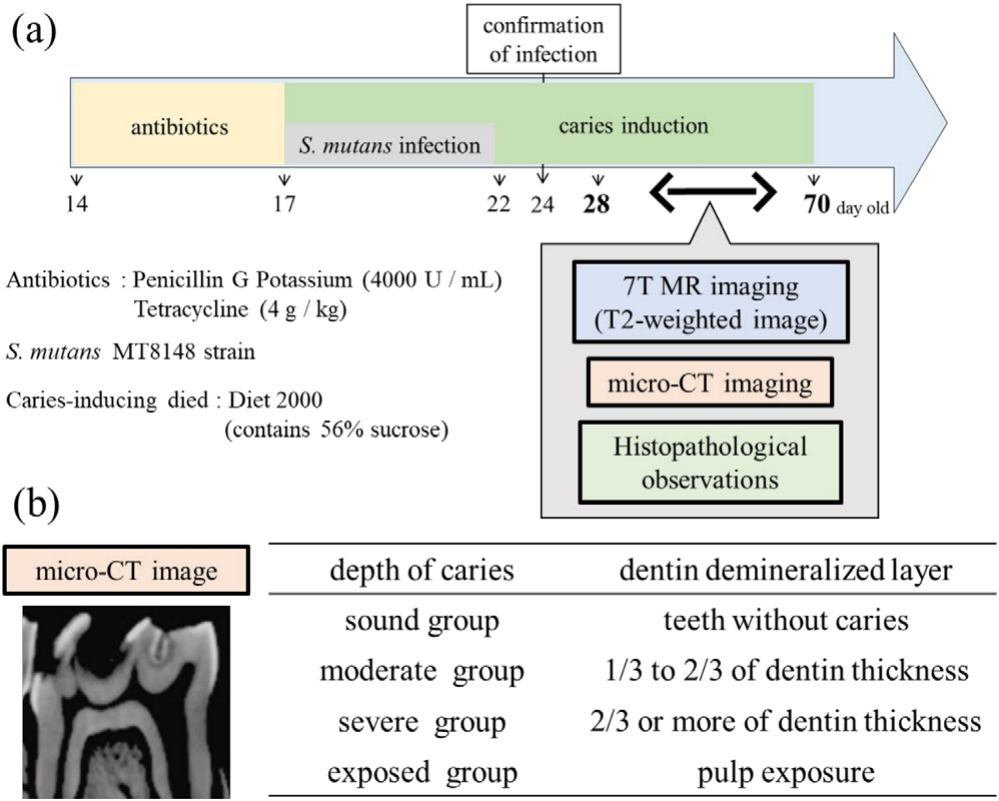
Overview of the rat caries‐induced pulpitis model. The timeline of the rat caries‐induced pulpitis model used in this study (a) and the classification of caries progression (b). All experiments were performed when the rats were between 28 and 70 days of age, and T2‐weighted imaging, micro‐CT imaging, and histopathological observations were performed as necessary (a). The degree of caries progression was classified as sound, moderate, severe, or exposed based on the micro‐CT images obtained at each time point (b). CT, computed tomography; MR, magnetic resonance.

Caries was induced in rat molars according to a previous report (Ooshima et al. 1988). The caries induction timeline and procedures are shown in Figure 1a. Fourteen‐day‐old male Sprague–Dawley (SD) rats (CLEA Japan Inc.) were administered antibiotics containing tetracycline (4 g/kg) (FUJIFILM Wako Pure Chemicals) and penicillin G potassium (4000 U/mL) (Meiji Seika Pharma) orally for 3 days to neutralize any nonspecific pathogens before caries induction. Thereafter, 17‐day‐old rats were orally infected using a saline solution of a *Streptococcus mutans* MT8148 suspension containing 1 × 10^8^ colony forming units/mL (200 μL) by a pipette for 5 days and fed ‘Diet 2000’, a cariogenic powdered diet (sucrose 56%, wheat flour 7%; CLEA Japan Inc. Infection) with *S. mutans* in oral mucosal swabs was confirmed at the age of 24 days. The rats were maintained for up to 70 days. Depending on the depth of the demineralized layer of the dentine, caries progression was classified as moderate, defined as demineralization that reached the middle third of the dentine; severe, defined as demineralization that reached the inner third of the dentine; or exposed, defined as pulp exposure by caries progression, as determined on micro‐CT images (SKYSCAN 1276, Bruker) (Figure 1b). Sound teeth without caries were used as controls (sound group). During the imaging procedure, a general anaesthetic mixture containing medetomidine hydrochloride (0.15 mg/kg) (Meiji Animal Health), midazolam (2 mg/kg) (Sandoz) and butorphanol tartrate (2.5 mg/kg) (Fujifilm Wako Pure Chemicals) was diluted with saline and administered intraperitoneally. The micro‐CT images were obtained at the following settings: maximum tube voltage 100 kVp, tube current 194 μA, slice width 40 μm and field of view 20 mm. Animals were monitored daily during caries induction for body weight, food and water intake, and signs of distress. Soft diet was provided if chewing difficulties were observed. No animals or data points were excluded from the analysis. All collected data were included in the final analysis. In each experiment, the number of animals used was planned to ensure that the study was scientifically valid while adhering to ethical principles by using the minimum number of animals required to obtain reliable results.

### 2.4 Sequential Observations Using MRI

Sequential observations of the caries‐derived pulpitis model were performed using MRI (Pharma Scan 70/16 US, Bruker). During caries induction, MRI and micro‐CT images were acquired once a week from the age of 28 to 70 days to sequentially observe the degree of caries progression and pulp condition in the same rat. T2‐weighted images were performed. The conditions of each imaging method are listed in Table 1.

**TABLE 1:** Imaging conditions used in this study for T2‐weighted and T2‐map images.

	T2‐weighted image	T2 map image
Sequence type	Fast spin echo	Fast spin echo
Voxel size	0.1 × 0.1 × 0.0363 mm^3^	0.125 × 0.125 × 0.0011 mm^3^
Matrix	320 × 320	256 × 256
Field of view	32 × 32 × 12 mm^3^	32 × 32 × 12 mm^3^
Number of slices	20	20
Slice thickness	0.3 mm	0.3 mm
Repetition time	3500 msec	2500 msec
Echo time	36 msec	9–108 msec (9 msec step 12 echo)
Bandwidth	122 Hz/pixel	122 Hz/pixel
Number of excitations	4	1
Echo train length	8	8
Time of acquisition	9 min 20 s	1 min 20 s

### 2.5 Searching ‘Internal Control’

To observe the dental pulp tissue using MRI, a suitable organ showing constant T2 values regardless of the age or an individual rat should be considered an internal control for magnetic resonance (MR) images of the rat dental pulp tissue. Two imaging methods were used in this study: T2‐weighted images, in which relative MR signal intensities were obtained, and T2‐map images, in which absolute T2 values were obtained. In this study, we defined the area with higher MR signal intensities than the surrounding area as ‘high MR signal area’ and the area with lower MR signal intensities as ‘low MR signal area’ on the T2‐weighted images. T2‐weighted and T2‐map images of 28‐day‐old male SD rats were obtained once a week using 7T MRI under general anaesthesia until they were 70 days old as was performed for CT. Four specimens were used. The obtained images were compared over time for the same rat. The major organs observed on T2‐weighted images, including the coronal pulp of rat teeth, were quantitatively evaluated using image analysis software (Image J Fiji, NIH, Bethesda) to obtain the T2 values of each organ for each day and rat. The region of interest (ROI) of each organ on the T2‐map image was manually set by referring to the same section in the T2‐weighted images, and the maximum luminance value in the ROI was determined according to a previous report (Juerchott et al. 2022). The T2 values of each organ obtained by quantitative evaluation were compared between the ages of 28 and 70 days to identify organs that could serve as an ‘internal control’ with nearly constant T2 values regardless of the day or the rat. The effect size used for the sample size calculation was estimated based on preliminary longitudinal MRI experiments evaluating variability in T2 values of candidate internal control organs across different ages and individuals. Considering an effect size of 1.2, a significance level of 5%, and test power of 95%, the calculated sample size was a minimum of four animals per group.

### 2.6 Quantification of the ‘MR Signal Ratio’ on T2‐Weighted Image

The ‘MR signal ratio’ in the coronal pulp just beneath the caries was quantified to compare the differences in pulp condition of each group. In this study, the ratio of the MR signal intensities of the coronal pulp and the internal control in the same section of the T2‐weighted image was defined as the ‘MR signal ratio’, which was calculated using the ImageJ Fiji software. The effect size used for the sample size calculation was estimated based on preliminary pilot experiments that evaluated differences in the MR signal ratio among representative experimental groups. Considering an effect size of 1.0, a significance level of 5%, and a test power of 95%, the sample size calculation indicated a minimum of six animals per group.

### 2.7 Histopathological Evaluation

The caries‐derived inflammatory pulp tissue was histopathologically evaluated. After the depth of the carious lesion and pulpal condition were analysed by micro‐CT and 7T MRI, animals were euthanized using an intraperitoneal overdose of sodium pentobarbital (Kyoritsu Seiyaku) and fixed with 4% paraformaldehyde solution (Nacalai Tesque), after which the maxillary and mandibular jaws, including the molars, were dissected, and soft tissues were removed. The samples were additionally fixed by immersion in the same fixative for 12 h at 4°C. Decalcification was performed by immersing the tissue in 0.1% ethylenediaminetetraacetic acid disodium salt and 4.3%–5.3% hydrochloric acid (KALKITOX, Fujifilm Wako Pure Chemicals) for 7 days at 4°C. The specimens were embedded in paraffin, and 6 μm sections were obtained using a rotary microtome (RM 2155; Leica). Haematoxylin & eosin (HE) staining (Fujifilm Wako Pure Chemicals, Mutoh Chemical, respectively) was performed to evaluate the general inflammatory condition. To evaluate the sequential inflammatory transition in the pulp tissue, immunohistochemical staining for CD43, a marker of inflammatory cells such as T lymphocytes and monocytes (Barran et al. 1997), CD68, a marker of macrophages (Allison et al. 2023), and Matrix Metalloproteinase 9 (MMP9), a candidate‐specific molecular marker for serious pulpal inflammation, was performed (Tsai et al. 2005; Sambandam and Neelakantan 2014; Mente et al. 2016; Rechenberg et al. 2016; Zanini et al. 2017; Chen et al. 2020; Al‐Natour et al. 2021; Kritikou et al. 2021; Fouad 2022). All sections were incubated in a DNA oven (MI‐100, Kurabo) at 56°C for 30 min before staining to prevent tissue detachment. After deparaffinization and rehydration, antigen retrieval was performed using a citrate buffer (10 mM citric acid, pH 6.0) and incubated in the oven at 78°C for 20 min. The sections were washed with phosphate‐buffered saline after the buffer returned to room temperature. The sections were incubated with 3% hydrogen peroxide (Kanto Chemical Inc.) that was diluted 10‐fold with methanol (Kanto Chemical Inc.) for endogenous peroxidase blocking. An ABC Kit (Vector Laboratories) was used for antigen detection according to the manufacturer's instructions. Mouse anti‐rat‐CD43 monoclonal antibody (ab22351; Abcam, Cambridge, UK) at a dilution of 1:500, rabbit anti‐rat‐CD68 polyclonal antibody (ab125212; Abcam) at a dilution of 1:400, and rabbit anti‐rat‐MMP9 polyclonal antibody (ab38898; Abcam) at a dilution of 1:250 were used as primary antibodies. After incubation with biotinylated secondary antibodies, the slides were incubated with avidin‐conjugated horseradish peroxidase. The horseradish peroxidase substrate was visualized using a DAB staining kit (Vector Laboratories, Burlingame, CA, USA). Nuclei were counterstained with Mayer's haematoxylin (Muto Pure Chemicals Co. Ltd.). All images were captured using a microscope (BZ‐X810; Keyence). All histological and immunohistochemical evaluations were performed by assessors who were blinded to the experimental group allocation (sound, moderate, severe and exposed) and the corresponding MRI findings. In cases of discrepant evaluations, the final decision was reached through discussion until consensus was achieved. In this study, the term ‘critical pulpal inflammation’ is used as an operational definition to describe localized pulp tissue exhibiting extremely serious inflammatory changes that, if preserved, induce serious inflammation or pulpal necrosis in the surrounding tissues and ultimately lead to apical periodontitis, but whose selective removal may facilitate healing of the remaining pulp tissue. This term does not imply global irreversibility of the pulp and is distinct from the traditional clinical diagnosis of ‘irreversible pulpitis’.

### 2.8 Evaluation of Artefacts in Materials for Pulpotomy

Magnetic susceptibility artefacts caused by various dental materials used in pulpotomy experiments were evaluated in MR images. The following materials were used for the pulpotomy experiments: ProRoot MTA (MTA) (Dentsply Sirona) as a pulp capping material, Clearfil Universal Bond Quick ER (Quick) (Kuraray Noritake Dental) as an adhesive material, and Clearfil Majesty ES Flow High (CR) (Kuraray Noritake Dental) as a composite resin for restoring the cavity. MTA was mixed according to the manufacturer's instructions, placed in a Teflon mould with a diameter of 3 mm and height of 10 mm, and tightly filled by pressure welding. Quick and CR were filled into the same‐sized mould sand and cured by light irradiation from two directions for 20 s each. The final cured product was placed in an incubator (ADVANTEC) at 37°C with 95% humidity for 24 h. An unfilled mould was used as the control. The samples were placed in 2.0‐mL microtubes (Eppendorf) and filled with distilled water. The T2‐weighted images of each microtube were obtained using the same MRI as used in the pulpotomy experiment. The imaging conditions for each imaging method were the same as those used in previous methods (Table 1).

### 2.9 Pulpotomy Procedure

General anaesthesia was administered to the rats using the same methods as described in the previous experiments. Local anaesthesia was additionally administered for the mandibular molars using a Xylocaine cartridge (Dentsply Sirona) containing lidocaine hydrochloride (20 mg/mL) and adrenaline (0.0125 mg/mL). The mandibular first molars of rats in the exposed group were isolated using a rubber dam and wiped with a cotton ball. The caries was then removed using a microexcavator (Sun Dental) and round bur (ISO #006, Dentsply Maillefer). A caries staining dye (Caries detector, Kuraray Noritake Dental) was used to confirm that the caries was completely removed. After removing the caries, a new sterile round bar was used to mechanically remove the coronal pulp corresponding to the low MR signal area. Micro‐CT images, the diameter of the round bar, and anatomic indicators, such as cusps, pulp horn, and cementoenamel junction, were used to determine the extent of pulpotomy. In addition, preoperative T2‐weighted images were used to identify low MR signal areas corresponding to severely inflamed pulp tissue. The spatial extent of the continuous low MR signal areas observed across serial MRI slices was integratively evaluated as a three‐dimensional spatial distribution. Based on this three‐dimensional estimation, the depth and extent of pulp tissue removal during pulpotomy were determined. The pulp surface was then chemically cleaned with 2.5% sodium hypochlorite to confirm haemostasis. The exposed pulp tissue was gently covered with MTA, which is a gold standard pulp‐capping material. The tooth surface was treated with Quick, and CR was used for the final restoration. Six weeks after treatment, T2‐weighted and micro‐CT images of the rat mandibular first molars were obtained, and histopathological evaluation was performed.

### 2.10 Statistics

Statistical significance was analysed using IBM SPSS Statistics for Windows, version 22 (IBM Corp., Armonk, NY, USA). An a priori power analysis was conducted using G*Power software (version 3.1.9.7; Heinrich Heine University Düsseldorf, Germany) to determine the minimum required sample size. Prior to applying parametric tests, the assumptions of normality and homogeneity of variances were assessed. The normality of data distribution was evaluated using the Shapiro–Wilk test, and homogeneity of variances was verified using Levene's test. As all datasets satisfied these assumptions, group comparisons were performed using one‐way analysis of variance followed by Tukey's post hoc test for multiple comparisons. *N*‐values and *p*‐values are shown in figure legends. All data are expressed as mean ± standard error of mean. A *p‐*value < 0.05 was considered statistically significant. Supporting data values are provided in the Data S1.

## 3 Results

### 3.1 Specific T2‐Weighted Images of Rat Molars at Different Stages of Caries Progression Can Be Obtained

**FIGURE 2:**
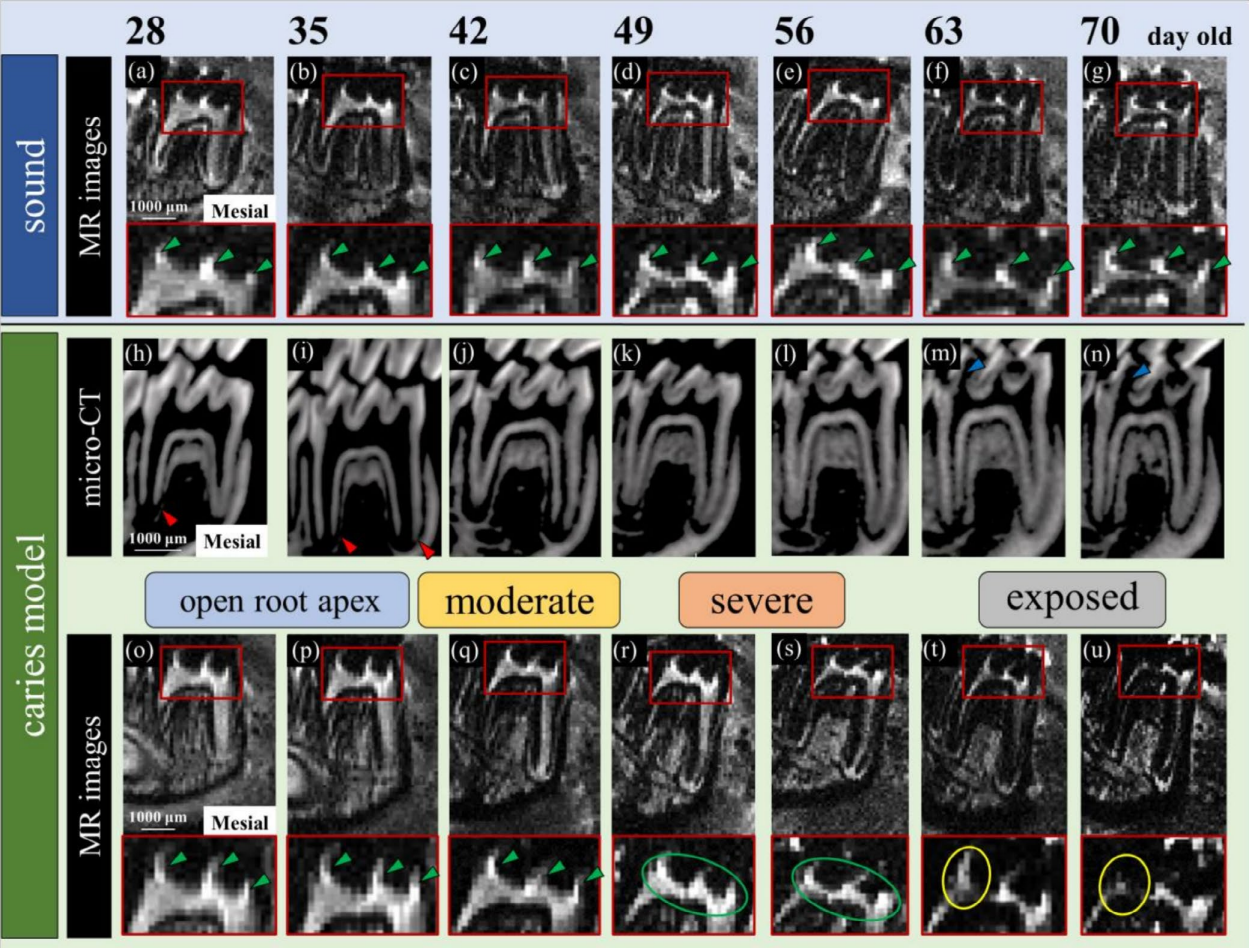
Micro‐CT and T2‐weighted images of caries‐induced pulpitis over time. T2‐weighted images of sound teeth (a–g) and micro‐CT (h–n) and T2‐weighted images (o–u) of the caries‐induced pulpitis model illustrate the caries progression and changes in the pulp and apical periodontal tissues over time. Red arrowheads: Open root apex, blue arrowheads: Pulp exposure, green arrowheads and circles: High MR signal area, yellow circles: Low MR signal area. CT, computed tomography; MR, magnetic resonance.

No modifications to the experimental protocols were necessary to prevent adverse health events, and no adverse events were observed throughout all experiments. In the sound group, a high MR signal area was observed in the pulp horn in almost all images of rats from the age of 28 to 70 days (Figure 2a–g). In the micro‐CT images of all groups, the root apices of the mandibular first molars were open, indicating incomplete root development at the age of 28 and 35 days (Figure 2h,i). The progression of caries in the mandibular first molars on micro‐CT images was evident as follows: moderate group at the age of 42 days (Figure 2j), severe group at the age of 49–56 days (Figure 2k,l), and exposed group at the age of 63–70 days (Figure 2m,n). In rats with initial carious lesions, when the root apex was open and caries had progressed to a lesser extent than that in the moderate group, a high MR signal area with a white depiction was observed in the pulp horn area (Figure 2o,p). The moderate group also showed a high MR signal in the pulp horn area, similar to that in the sound group at the same age (Figure 2q). In the severe group, a high MR signal area was observed in a wider area of the coronal pulp than in the moderate and sound groups (Figure 2r,s). In the exposed group, a low MR signal area was observed around the pulp exposure site (Figure 2t,u).

### 3.2 Deep Masseter Muscle Can Be an Internal Control

**FIGURE 3:**
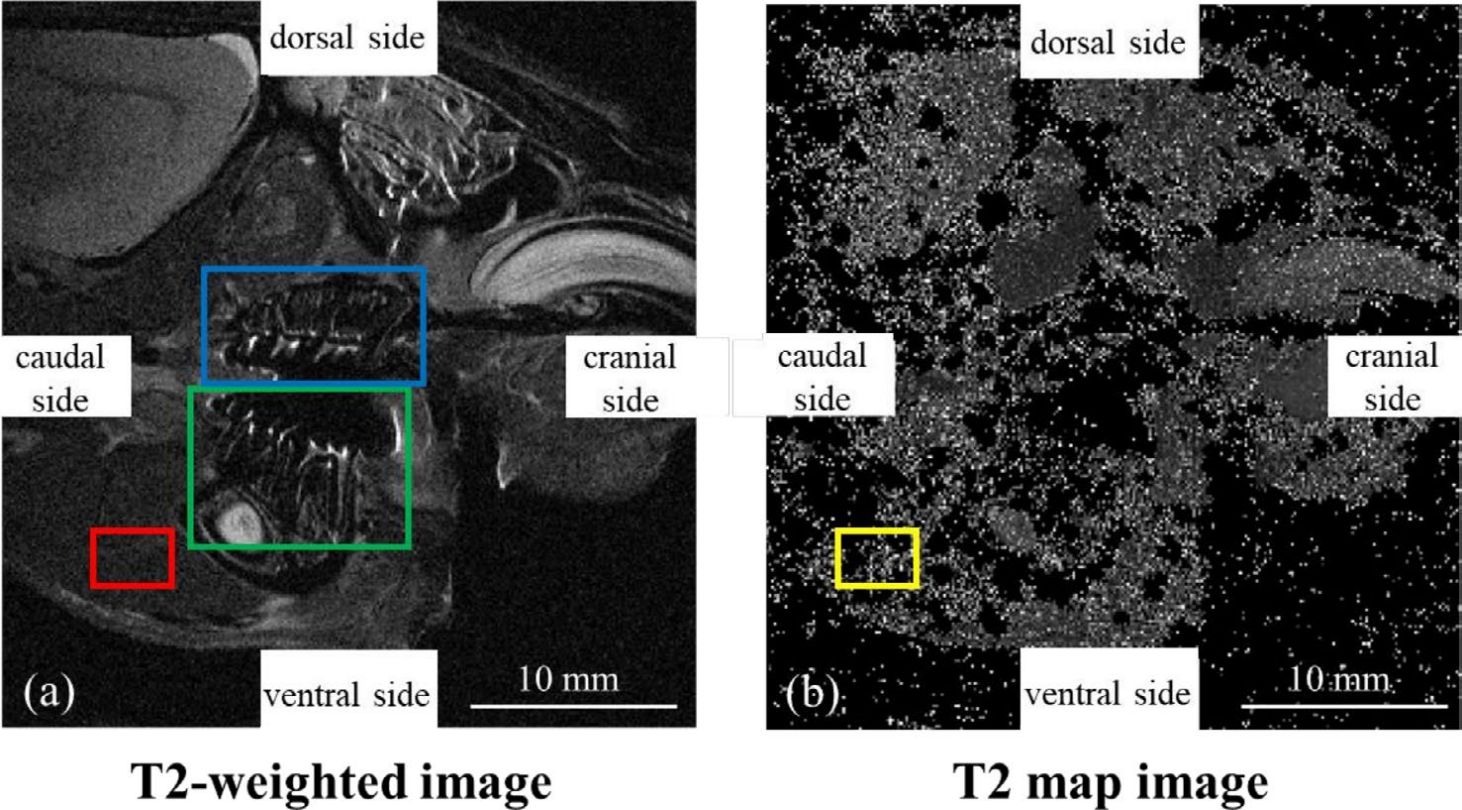
T2‐weighted and T2‐map images (sagittal section) of the head and neck of a 70‐day‐old rat. T2‐weighted (a) and T2‐map images (b) of the head and neck of a 70‐day‐old Sprague–Dawley rat (from the same sagittal section). Blue and green boxes: Maxillary and mandibular molars, respectively; red box in the T2‐weighted image: Deep masseter muscle; yellow box in the T2‐map image: Deep masseter muscle in the same area as the red box.

**FIGURE 4:**
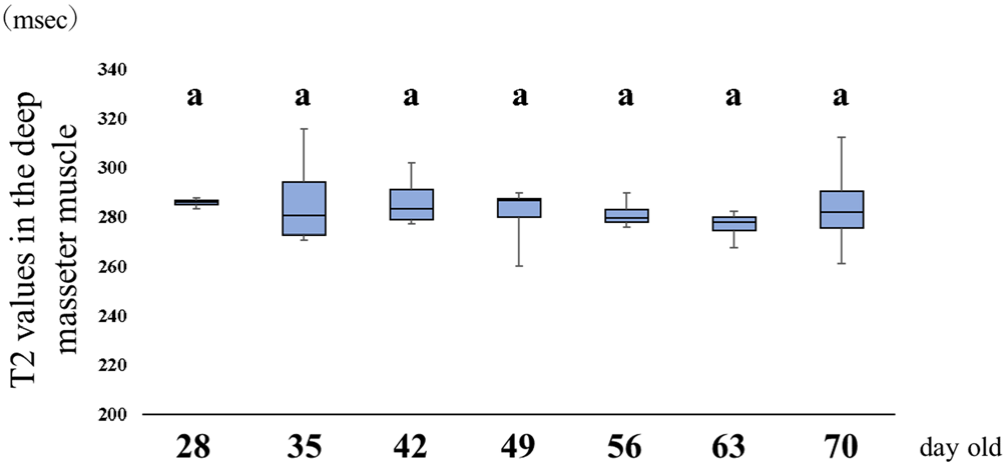
Comparison of T2 values of the deep masseter muscle on T2‐map images over time. T2 values of the masseter muscle in experimental Sprague–Dawley rats from the age of 28 to 70 days were compared. Boxes represent the IQR, with lines inside the boxes indicating the medians. Whiskers indicate the minimum and maximum values within 1.5 × IQR from the lower and upper quartiles, respectively. Same letters indicate no statistically significant difference (One‐way analysis of variance, Tukey post hoc test, *p* > 0.05, number of samples: 4). IQR, interquartile range.

Representative sagittal‐section MR images including the coronal pulp of T2‐weighted and T2‐map images of the same 70‐day‐old SD rat are shown in Figure 3. The T2 values of various organs on the T2‐map images were quantitatively evaluated using the ImageJ Fiji software and compared over time. The deep masseter muscle in the same sectional images, including the upper and lower coronal pulps, had almost constant T2 values without a statistical difference (*p* > 0.05), regardless of age and individual differences (Figure 4). Based on these results, we decided to compare and analyse the MR signal intensities obtained from T2‐weighted images using the deep masseter muscle as an internal control in subsequent experiments.

### 3.3 Exposed Group Showed a Significantly Lower MR Signal Ratio

**FIGURE 5:**
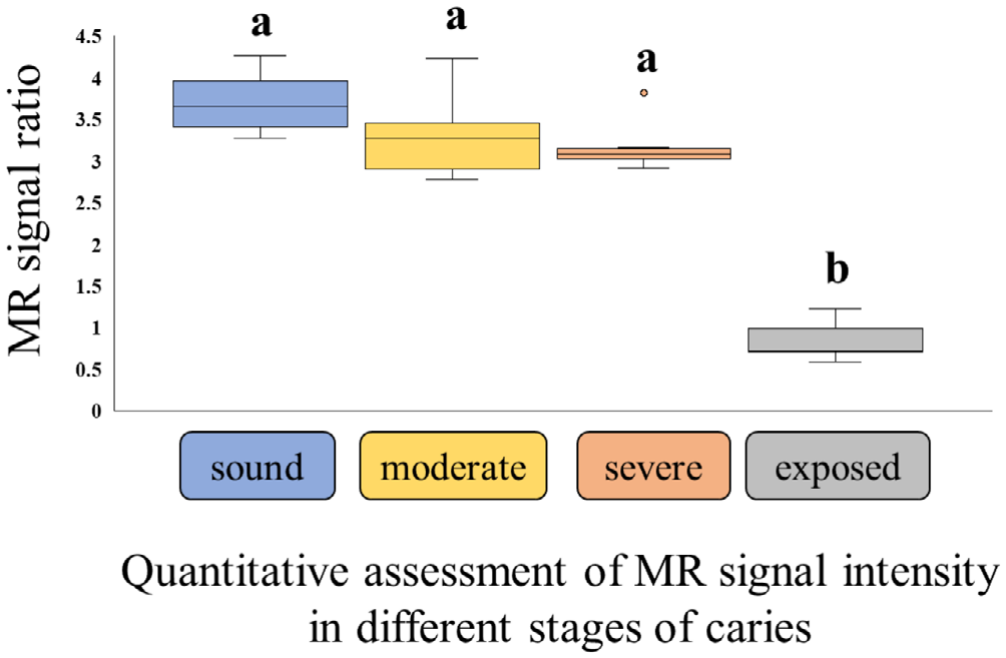
Quantitative evaluation of MR signal ratios at different stages of caries progression. The MR signal ratios of the coronal pulp just beneath the caries were quantitatively evaluated at each stage of caries progression. “The MR signal ratio” shown on the vertical axis represents the ratio of the MR signal intensities of the coronal pulp just beneath the caries and of the deep masseter muscle. Boxes represent the IQR, with lines inside the boxes indicating the medians. Whiskers indicate the minimum and maximum values within 1.5 × IQR from the lower and upper quartiles, respectively. Outliers are shown as individual points beyond the whiskers. Same letters indicate no statistically significant difference (One‐way analysis of variance, Tukey post hoc test, *p* < 0.05, number of samples: 6). IQR, interquartile range; MR, magnetic resonance.

The results of quantification of the MR signal ratios showed no significant differences between the sound, moderate, and severe groups (*p* > 0.05), while the exposed group showed a significantly lower MR signal ratio than the other groups (*p* < 0.05) (Figure 5).

### 3.4 Serious Inflammation Was Induced in the Low MR Signal Area

**FIGURE 6:**
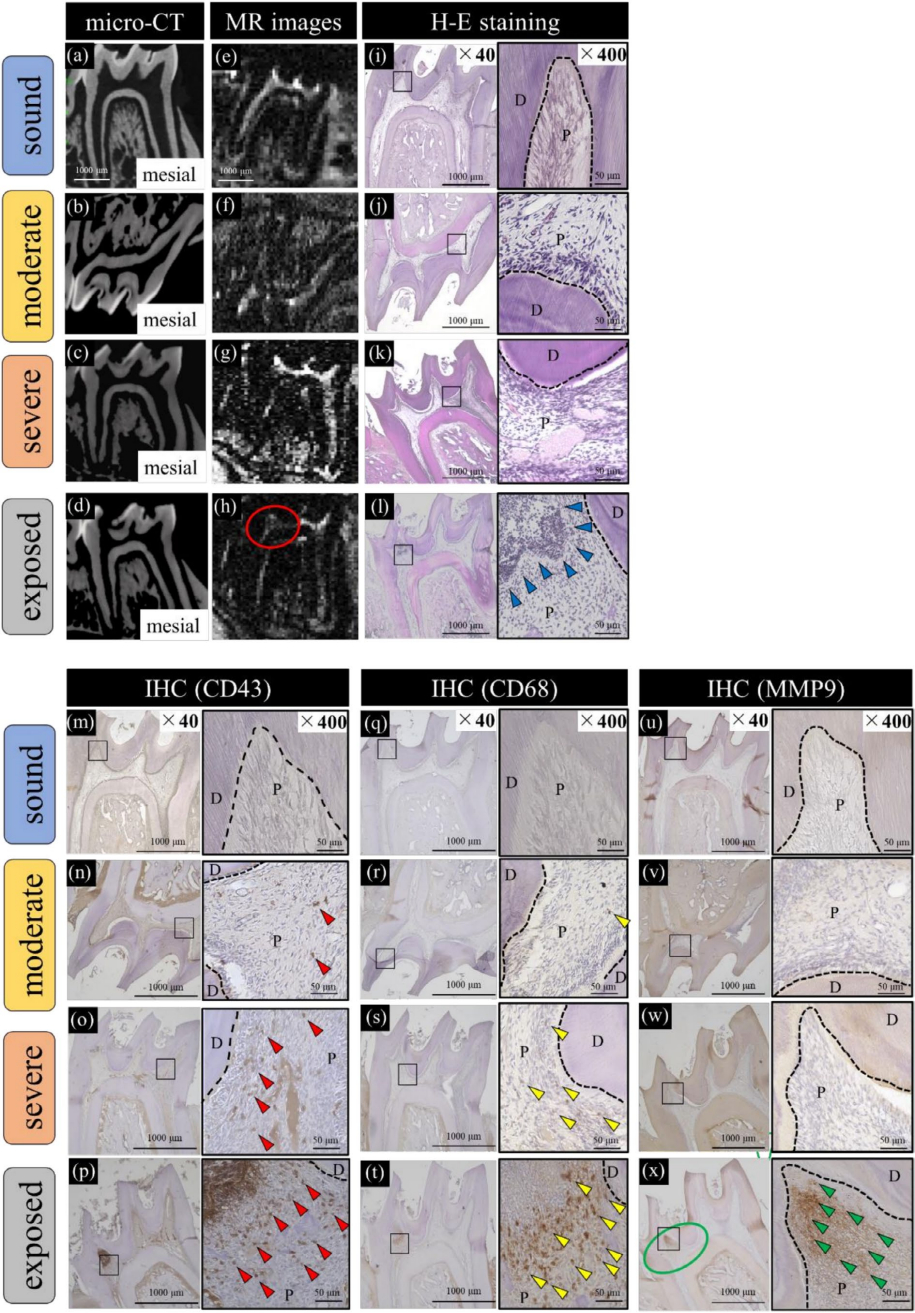
Micro‐CT images, T2‐weighted images, HE staining images, and immunohistochemical staining images against CD43, CD68, and MMP9 at each stage of caries progression. Micro‐CT images (a–d), T2‐weighted images (e–h), HE staining (i–l) images, and immunohistochemical staining images against CD43 (m–p), CD68 (q–t), and MMP9 (u–x) are shown for each stage of caries progression. Red circle: Low magnetic resonance signal area (h), blue arrowheads: Inflammatory cell‐like infiltration (l), red arrowheads: CD43‐positive cells (n–p), yellow arrowheads: CD63‐positive cells (r–t), green arrowheads: MMP9‐positive areas (x), green circle: MMP9‐positive area (x). D: Dentine, P: Pulp, Dashed line: Interface between pulp, dentine, alveolar bone, and periodontal ligament. CT, computed tomography; HE, haematoxylin & eosin; MMP9, Matrix Metalloproteinase 9.

Micro‐CT, T2‐weighted images, HE staining and immunohistochemical staining images at each stage of caries progression are shown in Figure 6. Micro‐CT and T2‐weighted images (Figure 6a–h) showed the same trend as that depicted in Figure 2. HE staining showed no inflammatory cell‐like infiltration or tertiary dentine formation in the pulp tissue and the presence of a well‐organized arrangement of odontoblasts in the sound group (Figure 6i). In the moderate group, limited inflammatory cell‐like infiltration was observed, along with tertiary dentine formation beneath the caries and a slight disarrangement of the odontoblasts (Figure 6j). In the severe group, a more severe inflammatory cell‐like infiltration, tertiary dentine formation beneath the caries, and a severe disarrangement of the odontoblasts were observed (Figure 6k). In the exposed group, tertiary dentine formation was sparse, odontoblasts beneath the pulp exposure had disappeared, and odontoblasts around the pulp exposure were extensively disorganized. Inflammatory cell‐like infiltration with a high nucleus‐to‐cytoplasm ratio was observed (Figure 6l).

Immunohistochemical staining revealed no expression of CD43 or CD68 in the sound group (Figure 6m,q). In the moderate group, some CD43‐ or CD68‐expressing cells were observed beneath the caries, coinciding with an area of high MR signal (Figure 6n,r). In the severe group, more CD43‐ or CD68‐expressing cells were observed in the pulp, along with an expansion of the high MR signal area (Figure 6o,s) than that in the moderate group. In the exposed group, CD43‐ or CD68‐expressing cells were observed in the entire pulp, and their number was greater around the area of pulp exposure (Figure 6p,t). In particular, these cells were accumulated beneath the area of pulp exposure. Immunohistochemical staining for MMP9, a candidate molecular marker for serious pulpal inflammation, showed no expression in the sound, moderate, or severe groups (Figure 6u–w). A strong MMP9 expression was observed only in the exposed group in the region where the MR signal intensity was diminished on the T2‐weighted images (Figure 6x).

### 3.5 No Material Used in the In Vivo Pulpotomy Experiments Induced Magnetic Susceptibility Artefacts

**FIGURE 7:**
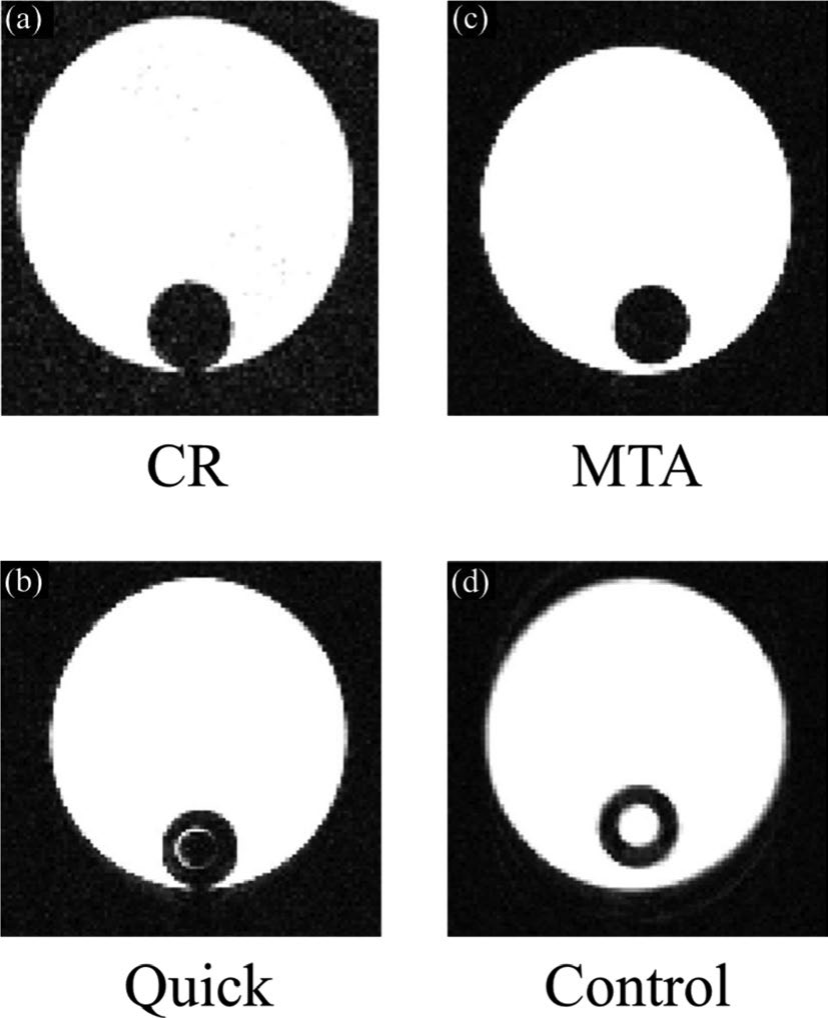
All materials used in the in vivo pulpotomy experiments did not induce magnetic susceptibility artefacts. T2‐weighted images of the dental materials used in the in vivo pulpotomy are shown (a–d). Each material was filled in a Teflon tube and placed in a microtube filled with distilled water. The white area in each figure indicates that the distilled water was imaged.

The results of magnetic susceptibility artefact analysis showed that no dental material used in the pulpotomy experiments caused any obvious magnetic susceptibility artefacts (Figure 7). Therefore, these materials were used in the pulpotomy experiments.

### 3.6 The Critical Pulpal Inflammation Zones Are Indicated by Significantly Low MR Signal Areas

**FIGURE 8:**
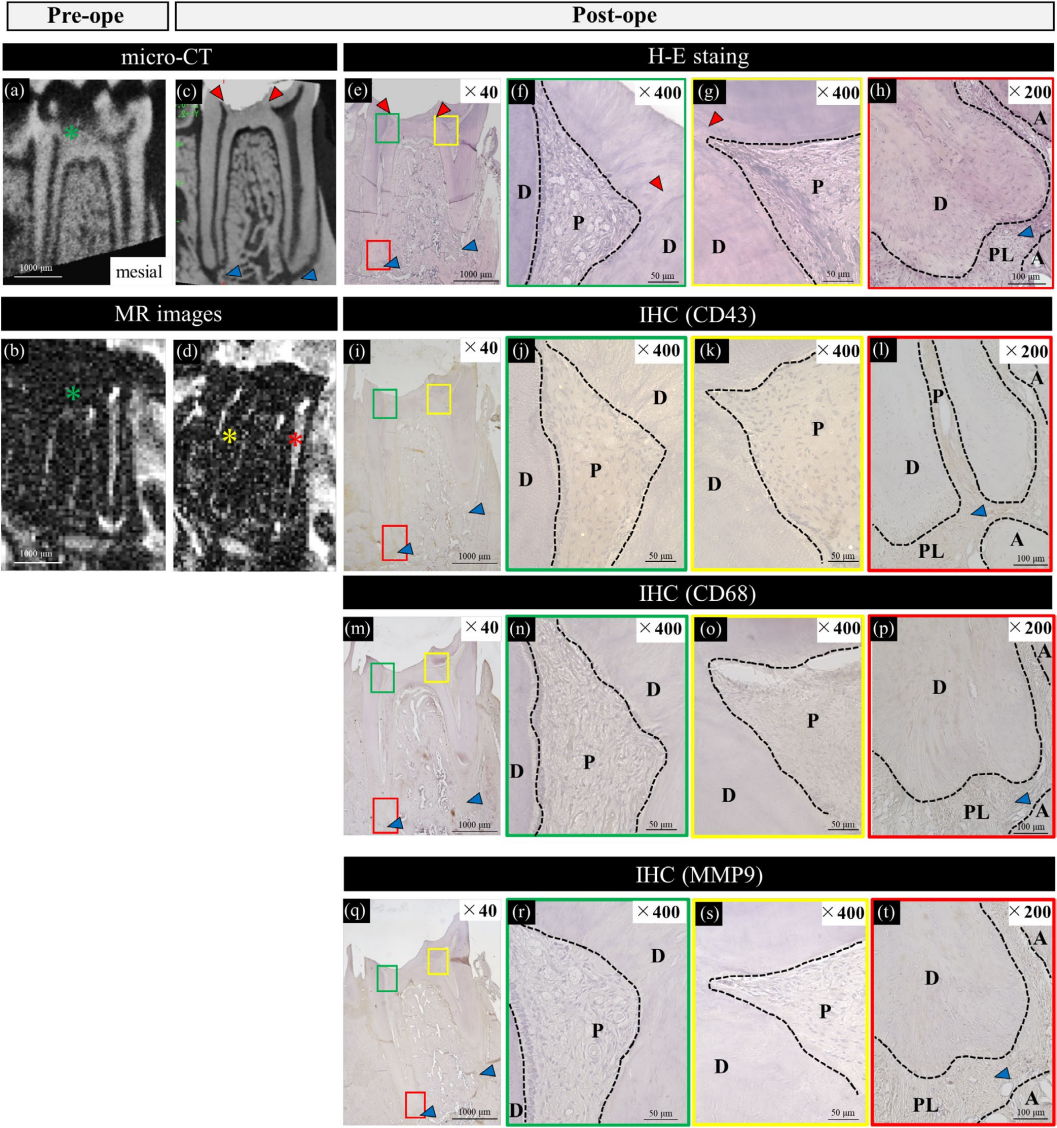
Micro‐CT images, T2‐weighted images, HE staining images, and immunohistochemical staining images against CD43, CD68, and MMP9 before and after pulpotomy. Micro‐CT images (a, c), T2‐weighted images (b, d), HE staining images (e–h), and immunohistochemical staining images against CD43 (i–l), CD68 (m–p), and MMP9 (q–t) are shown before and after the pulpotomy. Green asterisk: Exposed pulp cavity corresponding to the low MR signal area in micro‐CT image (a) or the low MR signal area in T2‐weighted image (b), red arrowheads: Formation of tertiary dentine (c, e–g), blue arrowheads: Translucent image (c) or the root apex without inflammatory reaction (e, h, i, l, m, p, q, t), yellow asterisk: Distal root without any sign of vitality after pulpotomy (D), red asterisk: Mesial root with signs of vitality after pulpotomy (D). D: Dentine, P: Pulp, A: Alveolar bone, PL: Periodontal ligament. Dashed line: Interface between the pulp, dentine, alveolar bone, and periodontal ligament. CT, computed tomography; HE, haematoxylin & eosin; MMP9, Matrix Metalloproteinase 9; MR, magnetic resonance.

In vivo pulpotomy experiments were performed to verify whether the serious pulpal inflammation evident as a low MR signal area was truly ‘critical pulpal inflammation’. Before pulpotomy, deep caries with an exposed pulp were induced in the objective tooth, which was confirmed by micro‐CT and T2‐weighted images revealing a low MR signal area around the pulp exposure (Figure 8a,b). Six weeks after pulpotomy, micro‐CT images revealed tertiary dentine formation in the area surrounding the area of pulpotomy and no radiolucency at the apex, suggesting that apical periodontitis was not induced and the pulp tissue was sound (Figure 8c). The T2‐weighted images showed a vital reaction in the mesial roots (Figure 8d). HE staining revealed satisfactory tertiary dentine formation beneath the pulpotomy material (Figure 8e–g). Further, no bone resorption or inflammatory cell‐like infiltration was evident in the root apex or pulp (Figure 8h). Neither CD43 nor CD68 cells were observed in the pulp or around the root apex after pulpotomy (Figure 8i–p). No MMP9 expression was observed in the pulp tissue or root apex (Figure 8q–t).

## 4 Discussion

In the caries‐affected pulp, various inflammatory reactions are triggered by the recognition of pathogens first by the outermost odontoblasts, followed by various immune and other cells (Farges et al. 2009; Duncan et al. 2019). When pulpal inflammation becomes severe due to caries progression, pressure in the pulp tissue increases due to effusion, causing ischemia and necrosis (Heyeraas and Berggreen 1999; Ricucci et al. 2019). Such inflammatory reactions in the pulp are first localized just beneath the caries and gradually spread to the entire pulp with caries progression (Bakhtiar et al. 2017; Ricucci et al. 2019). In this situation, before inflammation spreads to healthy areas, pulpotomy is considered an ideal treatment because a healthy pulp can be preserved by selectively removing the irreversibly inflamed pulp tissue (Duncan et al. 2019; Ricucci et al. 2019; American Association of Endodontists 2021). However, current pulp examination methods, which rely on the pain reported by patients, are highly subjective, and it is impossible to preoperatively determine the areas in the pulp tissue that should be removed (American Association of Endodontists 2021). Laser Doppler flowmetry (Miron et al. 2020) and pulse oximetry (Gopikrishna et al. 2007), which focus on blood flow in the pulp tissue and oxygen saturation in the blood, respectively, could be effective preoperative and objective diagnostic methods. These methods are expected to be accurate but are not deemed suitable for clinical application because of the need for a strictly standardized diagnostic environment (Schmitt et al. 1991; Schnettler and Wallace 1991; Kahan 1996; Leahy et al. 1999; Polat et al. 2004; Mejàre et al. 2012; Levin 2013; Alghaithy and Qualtrough 2017). MRI, which is now widely applied clinically, is attracting attention as a novel, objective, and preoperative diagnostic method that allows a more detailed observation of the pulp (Duncan et al. 2019; Ricucci et al. 2019; American Association of Endodontists 2021). Inflammatory reactions, such as effusion, affect the amount of fluid in the pulp tissue. Therefore, it may be appropriate to use T2‐weighted images, which can clearly detect differences in water content (Smith et al. 1985) and have been frequently used in previous reports on pulp observations (Gahleitner et al. 1999; Assaf et al. 2014, 2015; Iohara et al. 2016). However, there are no absolute values in T2‐weighted images, and only relative MR signal intensities are available. Therefore, T2‐weighted images alone cannot be used to directly compare images of different sections of the same rat or different rats. Accordingly, in this study, both T2‐weighted and T2‐map images, for which absolute T2 values can be obtained, were obtained simultaneously from the same rats. The deep masseter muscle showed nearly constant T2 values regardless of age or individual differences (Figure 4). Therefore, we used this organ as an internal control for the calibration of T2‐weighted images in subsequent experiments.

To elucidate the pathogenesis of caries‐derived pulpitis, caries progression in rat mandibular first molars and pulpal conditions were monitored sequentially. Sequential observations of the same rats showed that the root apices of 28‐ and 35‐day‐old rats were open in both the caries‐derived and sound groups (Figure 2a,b). Root apex formation of mandibular first molars in SD rats is complete at the age of approximately 35 days (Xu et al. 2011), which is consistent with the results of this study. Because the pulpal immune response of teeth with incomplete root formation may differ from that of mature permanent teeth, we focused on teeth of 42‐day‐old rats, when the root formation is complete, in subsequent experiments. In the quantitative evaluation of the MR signal ratio, the maximum brightness value for each ROI was used as the MR signal intensity, in accordance with a previous report (Juerchott et al. 2022). This is because the pulp is surrounded by dentine and enamel, which has a low water content and an extremely low MR value (Juerchott et al. 2022). Specifically, if the hard tissue surrounding the pulp is even partially included in the ROI, the minimum or average brightness values could be significantly affected, making it difficult to obtain accurate brightness values for the pulp tissue. However, a previous study reported that the maximum value was not affected by the surrounding hard tissues with extremely low brightness values and that accurate results could be obtained (Juerchott et al. 2022). Therefore, in this study, the maximum brightness value for each ROI was used as the MR signal intensity of the pulp.

Sequential changes in the pulp on T2‐weighted images were detected in the same rats. Specific T2‐weighted images were obtained according to the caries progression stage (Figure 2). This indicated that MRI might be a useful tool for non‐invasive and sequential assessment of the pulpal condition. Different pathological mechanisms operate in the pulp at each stage of caries progression. Therefore, histopathological observations were performed at each stage of caries progression to elucidate the detailed phenomenon occurring in the pulp tissue for comparison with the MRI results of the subsequent experiments.

Inflammatory cells, such as T lymphocytes, neutrophils, monocytes and macrophages, infiltrate the pulp tissue in teeth with pulpitis (Izumi et al. 1995; Yoshiba et al. 2003; Nakanishi et al. 2005; Novak and Koh 2013). In this study, we evaluated pulp inflammatory reactions using immunohistochemistry with CD43 (Barran et al. 1997), a marker of inflammatory cells such as T lymphocytes and monocytes, and CD68 (Allison et al. 2023), a marker of macrophages. In addition, specific indicators of pivotal pulpal inflammation were included. Therefore, we used a previously reported candidate marker for serious pulpal inflammation. Various specific molecular biomarkers for serious pulpal inflammation have been assessed, and inflammatory cytokines and proteolytic enzymes, such as interleukin‐1β, interleukin‐6, tumour necrosis factor‐α, and MMP9, have been focussed on as candidates (Elsalhy et al. 2013; Chen et al. 2020; Kritikou et al. 2021; Wu et al. 2022). Among these, MMP9 has been reported more frequently than the other molecules in clinical samples (Tsai et al. 2005; Sambandam and Neelakantan 2014; Mente et al. 2016; Rechenberg et al. 2016; Zanini et al. 2017; Chen et al. 2020; Al‐Natour et al. 2021; Kritikou et al. 2021; Fouad 2022). Therefore, in this study, we used MMP9 as an indicator of serious pulpal inflammation. In general, a high MR signal intensity on T2‐weighted images indicates the presence of more protons in water in that region (Sanjay and Ernest 2018). Therefore, the higher MR signal intensity in an organ compared with that in a healthy area could be caused by an increase in water content caused by inflammation, oedema or cancer (Sanjay and Ernest 2018).

In the sound group, although a high MR signal was observed in the pulp horn, no inflammatory cells were observed (Figure 6e,i,m,q,u). This strong signal might be related to cuspal attrition in rats, as evident in the micro‐CT images (Figure 6a). In other words, the pulp horn just beneath attrition is constantly exposed to external stimuli, resulting in an active defence reaction of the pulp to form acquired tertiary dentine. Further, the water content increases due to the influence of cells that were not detected by the immunohistochemical staining performed in this study. This indicates that a high MR signal area does not always indicate the presence of inflammation when MRI is used to diagnose pulpitis. Therefore, when MRI is used to diagnose pulpitis, this information should be considered in the presence of inflammation.

Histopathological observations of the moderate and severe groups revealed tertiary dentine formation and disorganized arrangement of the odontoblast layer (Figure 6j,k), which may be due to the defence activities of the pulp tissue against caries invasion. Immunohistochemical staining against CD43 and CD68 revealed that the inflammatory cell‐like infiltrate expanded to include the entire coronal pulp as the caries progressed from the moderate to severe group, and these results coincided with the spread of the high MR signal area (Figure 6f,g,n,o,r,s,v,w). Inflammatory cells, such as T lymphocytes and macrophages expressing CD43 and CD68, are observed in the pulp tissue just beneath the caries as pulpitis progresses (Izumi et al. 1995; Yoshiba et al. 2003; Nakanishi et al. 2005; Novak and Koh 2013). This suggests that increased vascular permeability and spread of effusion in pulpal inflammation are induced just beneath the caries, and a high MR signal is observed owing to the increased water content in the same area that was assessed using immunohistochemical staining. These results are consistent with previous reports that inflammatory reactions in the pulp are initiated close to the carious lesion and gradually spread to a wider area (Bakhtiar et al. 2017; Ricucci et al. 2019). In contrast, MMP9 expressions were not observed at these stages (Figure 6v,w), indicating that these pulpitis stages may be considered relatively mild. Additionally, quantitative evaluation of the MR signal ratio in the moderate and severe groups did not show significantly different results (Figure 5). These results indicate that it might be difficult to distinguish between the degrees of inflammation in the moderate and severe groups using MRI. Based on these findings, MRI may be useful for estimating the spatial extent of inflammation in relatively mild pulpitis; however, differential diagnosis of inflammatory severity remains challenging. To address this issue, further investigations are required. Recent studies have reported that the use of improved receiving coils enables acquisition of higher‐resolution MR images of the dental pulp (Zaike et al. 2025). In future studies, the application of such advanced coils, together with the acquisition of T2‐weighted images at shorter intervals and improvement of MR image resolution for more accurate definition of the ROI, may allow a more detailed elucidation of the pathophysiology of caries‐derived pulpitis.

In the T2‐weighted images of the exposed group, a low MR signal area was observed around the pulp exposure site. This is consistent with a previous report on the relationship between the stage of human caries progression and T2 values, which showed that the pulp affected by deep caries had lower T2 values than the healthy pulp (Cankar et al. 2020). In addition, this pulp showed a significant infiltration of CD43‐ and CD68‐positive cells (Figure 6p,l), and MMP9 expression, which was not observed in the other groups, almost always coincident with the low MR signal area (Figure 6x). These results suggest that a more severe inflammatory reaction occurred in the low MR signal area. Generally, the inflamed area is signified by a high MR signal area on T2‐weighted images (Sanjay and Ernest 2018); however, the results of this study were different. A decrease in free water causes low‐signal intensity on T2‐weighted images (Carpentieri‐Primo et al. 2024), likely driven by histopathological changes associated with severe inflammation, including tissue necrosis, intrapulpal haemorrhage, fibrin deposition, and the aggregation of inflammatory cells. Such changes are known to reduce free water mobility and shorten transverse relaxation times, which may contribute to the appearance of low T2 signal areas in severely inflamed pulp tissues (Curvo‐Semedo et al. 2010; Zimny et al. 2015; Kang et al. 2023; Carpentieri‐Primo et al. 2024). However, the detailed mechanisms underlying the development of low MR signal areas in more severe pulpitis remain to be fully elucidated and require further investigation.

In addition, the low MR signal area in the exposed group might be associated with more serious inflammation, with apparent MMP9 expression, than in the other groups, which might correspond to critical inflammatory zones. To verify whether critical pulpal inflammation could actually occur in this area, we physically removed this area by pulpotomy and observed the inflammatory reaction and wound healing process in the remaining pulp tissue.

Some dental materials cause artefacts on MRI (Bohner et al. 2022). However, there are no reports on the materials used in pulpotomy experiments that cause artefacts. Before performing the pulpotomy experiments, artefacts from the dental materials used in the pulpotomy experiments were investigated, and we found that none of the materials caused susceptibility artefacts (Figure 7). Differences in magnetic susceptibility are the primary cause of susceptibility artefacts (Graves and Mitchell 2013). Based on these results, the cause of artefacts was not attributed to these materials.

In the pulpotomy experiment in which the low MR signal area was physically removed, normal wound healing was induced in the remaining pulp tissue. This result was confirmed by tertiary dentine formation and the lack of inflammatory cell infiltrate and MMP9 expression in the pulp (Figure 8e–g,i–k,m–o,q–s). This is consistent with a previous report that after appropriate caries removal, the disappearance of inflammatory cells, such as T lymphocytes, that had previously infiltrated the pulp, induced wound healing (Yoshiba et al. 2003). In addition, these results demonstrate the high reliability of MMP9 as a biomarker for critical pulpal inflammation. However, whether MMP9 can identify the boundary between critical pulpal inflammation and pulp tissue with healing potential remains unclear. A more comprehensive analysis is necessary to identify better markers. Furthermore, long‐term outcomes after pulpotomy were not evaluated in this study. Long‐term prognosis following pulpotomy is influenced by multiple factors, including the accuracy of preoperative diagnosis and coronal sealing quality and microleakage (Aguilar and Linsuwanont 2011; Asgary and Nosrat 2025). In this context, the extent to which MRI‐based preoperative diagnosis may influence long‐term outcomes warrants further detailed investigation.

T2‐weighted images obtained after pulpotomy showed a high MR signal in the mesial root (Figure 8d). The MR signal in the pulp indicates pulp vitality (Iohara et al. 2016; Nakashima et al. 2017); therefore, the pulp in the mesial root could be vital after pulpotomy. In contrast, no MR signal was observed in the distal root after pulpotomy in the same section, and the appearance indicated inflammation or necrosis (Figure 8d). This might be because the distal root canal could not be visualized in a single‐slice image because the slice thickness of the T2‐weighted image was 300 μm, whereas the diameter of the distal root canal is approximately 100 μm. In histopathological observations, no obvious inflammatory reaction in the pulp tissue of the distal root, tertiary dentine formation, or radiolucency at the apex on micro‐CT was observed after pulpotomy (Figure 8c,e,f,i,l–n,q,r), suggesting that the distal root canal could maintain vitality, even though it was not depicted on the T2‐weighted images.

In this study, we used 7T MRI, an ultra‐high magnetic field MRI, to observe the pathophysiology of rat pulp, but this advanced MRI is currently unavailable for clinical use in some countries. Nevertheless, sufficiently accurate images can be obtained by 3T MRI, which is widely used in many countries, using an appropriate receiving coil adapted to human dentition (Juerchott et al. 2022). However, the introduction of MRI into dental practice is still associated with several unresolved challenges, including high costs and prolonged acquisition times, which hinder its routine use (Idiyatullin et al. 2011; Flügge et al. 2023). Future improvements in MRI hardware and imaging protocols are expected to help overcome these limitations. Furthermore, at present, MRI has several contraindications, such as its inability to be used in patients with pacemakers (Dill 2008), and certain drawbacks, including degradation of image quality due to magnetic susceptibility artefacts when metallic restorations are present in the oral cavity (Mathew and Maller 2013; Bohner et al. 2022). These issues should be carefully considered during implementation of MRI‐based diagnosis of pulpitis in the future. This study should be regarded as a non‐clinical proof‐of‐concept investigation demonstrating the feasibility of MRI‐based assessment of caries‐derived pulpitis under experimental conditions. The results of this study provide a foundation for future research on MRI‐based approaches for the diagnosis of pulpitis and may contribute to the eventual clinical translation of MRI for pulpal diagnosis.

## 5 Conclusion

This study demonstrated that MRI could be used to selectively and preoperatively diagnose caries‐derived critical pulpal inflammation, establishing the basis for a novel, non‐invasive, and quantitative diagnostic method for pulpitis. In addition to preoperative diagnosis, the establishment of intraoperative diagnostic methods is important to achieve a high success rate of pulp conservation therapy, including pulpotomy. If a specific reliable marker can be identified more accurately, an intraoperative diagnosis of pulpitis would be possible, and the predictability and success rate of vital pulp therapy will be improved. This study shows that MMP9 is a reliable marker of critical pulpal inflammation. However, it is necessary to verify whether MMP9 can identify the boundary between critical pulpal inflammation and pulp tissue with healing potential. In the future, we will investigate the potential of MMP9 in detail and perform a comprehensive analysis of the low MR signal areas to identify other specific markers of critical pulpal inflammation.

## Author Contributions

K.M., M.O. and Y.T. performed the experiments. K.M. performed most of the experiments, and K.M., M.O. and Y.T. helped develop the protocol and provided technical assistance. M.W., H.H. and K.N. contributed to data acquisition, analysis and interpretation in most experiments and provided technical assistance in the pulpotomy experiments. S.S. performed MRI of all samples using 7T MRI and provided advice on the project design and data interpretation. K.M. performed statistical analyses. K.M., Y.T., and M.H. wrote the manuscript. Y.T. revised the manuscript. All authors gave their final approval and agreed to be accountable for all aspects of this work.

## Ethics Statement

The manuscript of this animal study has been written according to Preferred Reporting Items for Animal studies in Endodontology (PRIASE) 2021 guidelines (Nagendrababu et al. 2021). All animal studies were approved by the Institutional Animal Care and Use Committee of Graduate School of Dentistry, the University of Osaka (No. R‐01‐017‐0).

## Conflicts of Interest

The authors declare no conflicts of interest.

## Supporting information


**Data S1:** Supporting data values file.

**Data S2:** PRIASE 2021 checklist.

## Data Availability

The values for all the data points in the graphs are reported in the Data S1.
